# Surgical management of traumatic posterior hip dislocation associated with ipsilateral femoral shaft fracture in a child via the anterior approach: a case report and literature review

**DOI:** 10.3389/fped.2026.1822934

**Published:** 2026-04-10

**Authors:** Lei Xie, Honghong Pei, Qichao Ma

**Affiliations:** 1 Department of Pediatric Orthopedics, Bozhou People’s Hospital, Bozhou, Anhui, China; 2 Department of Orthopedics, Shanghai Children’s Hospital, School of Medicine, Shanghai Jiao Tong University, Shanghai, China

**Keywords:** avascular necrosis, femoral shaft fracture, floating hip variant, pediatric hip dislocation, smith-Petersen approach

## Abstract

**Background:**

Traumatic hip dislocation combined with an ipsilateral femoral shaft fracture is an extremely rare and severe injury, particularly in children, with an annual incidence of traumatic joint dislocations around 1 per 100,000 in pediatric populations. The absence of a femoral lever arm complicates reduction, often requiring open techniques. While posterior approaches are traditional for posterior dislocations, they risk further compromising the femoral head's blood supply in pediatric patients.

**Case presentation:**

We report a 10-year-old boy who sustained a high-energy injury leading to posterior dislocation of the right hip and an ipsilateral displaced femoral shaft fracture. Treatment involved open reduction via the anterior Smith-Petersen (S-P) approach to safeguard posterior vascularity, combined with lateral plating of the femur. At 18-month follow-up, the patient achieved full weight-bearing, complete range of motion, solid femoral union, a congruent hip joint, and no evidence of avascular necrosis (AVN) or leg-length discrepancy.

**Conclusion:**

In pediatric cases of this complex injury, anterior open reduction paired with rigid femoral fixation offers a safe and effective option. It enables anatomic reduction while potentially reducing iatrogenic damage to the medial circumflex femoral artery, thereby lowering AVN risk, which can increase over fivefold with delays beyond 12 h.

## Introduction

Traumatic hip dislocation with an ipsilateral femoral shaft fracture represents a rare and challenging injury pattern, typically resulting from high-energy trauma such as motor vehicle accidents or falls from height (1–3). Often termed a “floating hip” variant, this combination is uncommon in children due to the acetabulum's plasticity but can occur with substantial force (3–5). The orthopedic priority is timely hip reduction—ideally within 6 h—to mitigate avascular necrosis (AVN) risk, which rises significantly with delays beyond 12 h (1, 2, 6, 7). However, the femoral fracture disrupts the lever arm essential for closed reduction maneuvers, rendering them ineffective or risky and potentially causing iatrogenic physeal injury (3, 8–10).

In children, preserving femoral head vascularity and the proximal physis is critical. Forceful closed attempts can lead to iatrogenic harm, making open reduction frequently necessary when closed methods fail (5, 11, 12). For posterior dislocations, the posterior Kocher-Langenbeck approach is traditionally favored (4, 13), yet this risks a “second hit” to already compromised posterior tissues, potentially jeopardizing the medial circumflex femoral artery (MCFA)—the dominant pediatric femoral head blood supply—and increasing AVN risk (14). Conversely, anterior approaches such as the Smith–Petersen (S–P) approach offer an alternative access route.

In children, anterior approaches are particularly advantageous due to greater tissue elasticity and healing potential. Critically, pediatric vascular anatomy differs from adults: the medial circumflex femoral artery (MCFA) is the dominant femoral head blood supply in children and is particularly vulnerable to posterior dissection during the Kocher–Langenbeck approach, making anterior preservation strategies biologically compelling in this age group (14). While anterior approaches are well-documented for anterior dislocations in children, their application to posterior dislocations constitutes a significant knowledge gap. The S–P approach may offer distinct vascular preservation advantages precisely when posterior soft-tissue disruption has already occurred. This case represents, to our knowledge, the first description of the S–P approach for traumatic posterior hip dislocation with ipsilateral femoral shaft fracture in a child, highlighting the rationale for this vascular-preserving strategy and reviewing pertinent literature to address this lacuna.

## Case presentation

### History and examination

A 10-year-old boy was transferred to our institution 25 h post-injury following initial management at a referring hospital. The injury occurred during a motor vehicle accident where the vehicle, in which he was a rear-seat passenger, struck a railing and submerged in a river. At the referring institution, skin traction was applied for 24 h with attempted closed reduction, which proved unsuccessful due to the absent femoral lever arm. Given the complexity and resource limitations, transfer was arranged.

Upon arrival at our emergency department, examination revealed: the right lower extremity positioned in internal rotation; marked shortening of approximately 4 cm compared to the contralateral side; the dislocated femoral head was palpable posteriorly in the right hip region with significant tenderness; and restricted active and passive range of motion of the right hip. The right knee, ankle, and toes demonstrated full active motion. The dorsalis pedis pulse was palpable, and sensation was intact in all distributions. The contralateral limb and pelvis were unremarkable. No associated head, chest, or abdominal injuries were identified on systematic trauma survey.

### Imaging

Initial evaluation at the referring institution included anteroposterior radiography of the right hip and femur, with images available only as a single digital photograph captured on a mobile device (quality limited by non-diagnostic acquisition). These demonstrated posterior hip dislocation and displaced femoral shaft fracture, prompting transfer. Dedicated orthogonal radiographs were not obtained at our institution given the elapsed 24-h interval and urgency of Computed tomography (CT) -based surgical planning.

**Figure 1:**
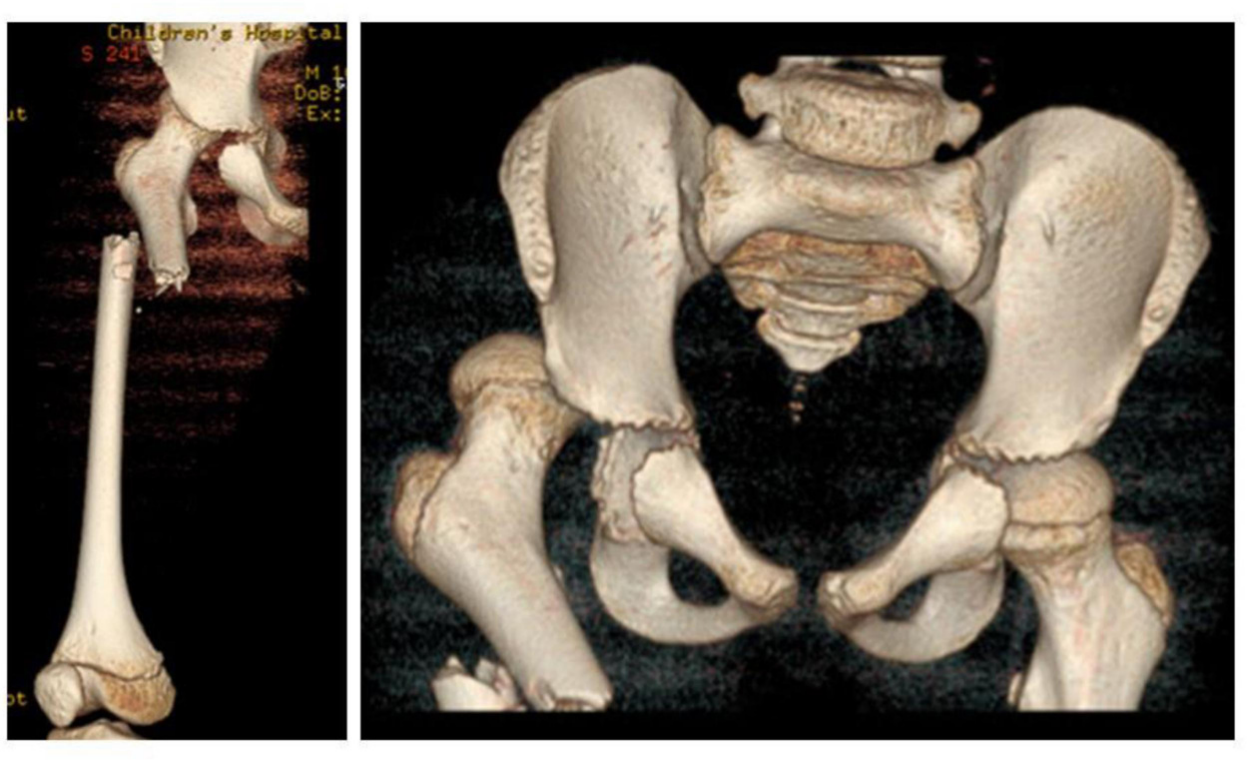
Computed tomography(CT) showed that a displaced fracture of the proximal-middle segment of the right femoral shaft and a posterior dislocation of the right hip joint.

CT with 3D reconstruction (Figure 1) provided definitive characterization: posterior hip dislocation without femoral head fracture; displaced short oblique fracture of the proximal-middle femoral shaft with 100% displacement and 15° varus angulation; moderate hemarthrosis with intra-articular gas; posteroinferior labral tear with capsular stripping; no loose osteochondral fragments; and intact posterior acetabular wall. These findings—particularly the labral pathology invisible to plain radiography—directly guided the decision for anterior open reduction.

### Surgical management

Upon admission (25 h post-injury), the patient was optimized for surgery: hemoglobin stabilized at 10.8 g/dL with cross-matched blood available, cefuroxime prophylaxis administered, and nil per os status maintained. Given the failed closed reduction at the referring institution and elapsed time, repeat manipulation was avoided to prevent additional soft-tissue trauma. Surgery proceeded under general anesthesia in supine position using a two-stage “femur-first” approach:

Stage 1: Femoral Shaft Fixation. A 10-cm lateral incision exposed a short oblique, comminuted fracture with 2 cm × 1 cm cortical deficiency. Following hematoma evacuation, anatomical reduction was achieved under direct vision. A cortical autograft fashioned from locally harvested comminuted fragments and cortical shavings was impacted into the defect to restore continuity and enhance biological healing. Temporary Kirschner wire stabilization preceded application of a 120° pediatric hip plate (PHP) with 7 screws. Fluoroscopy confirmed anatomical alignment and stable fixation.

Stage 2: Open Hip Reduction via Smith-Petersen Approach. With the femur now providing stable leverage, a separate anterior incision developed the tensor fasciae latae-sartorius interval. The rectus femoris was retracted without detachment to preserve ascending branch vessels. T-shaped capsulotomy enabled direct visualization: the femoral head was posterior to the acetabulum with posteroinferior labral tear and capsular stripping.

Traction applied through the fixed femoral plate (used as joystick) with counter-traction via ipsilateral iliac wing; the femoral head was gently levered into position using a blunt bone tamp through the anterior capsulotomy, maintaining 90° flexion and neutral rotation. A 1.6 mm Kirschner wire drilled into subchondral bone demonstrated active bleeding within 3 s, confirming preserved perfusion. The avulsed ligamentum teres was excised; labrum debrided and repaired with 2-0 Vicryl sutures. Stability confirmed through full range of motion without subluxation. Capsulorrhaphy with interrupted 2-0 Vicryl completed the repair. Intraoperative blood loss was 150 mL; the patient received 150 mL red blood cell transfusion.

### Postoperative course

A hip spica cast was applied for 4 weeks. Rationale: (1) protection of both femoral osteosynthesis and capsulorrhaphy; (2) unreliable compliance with protected weight-bearing in a 10.9-year-old; (3) high-energy injury with labral repair requiring joint rest.

Rehabilitation included non-weight-bearing for 4 weeks, progressing to partial weight-bearing at 4–6 weeks and full at 8–12 weeks, with physical therapy focusing on range of motion, strengthening, and gait training. Multimodal analgesia and infection prophylaxis were employed.

### Outcome

**Figure 2:**
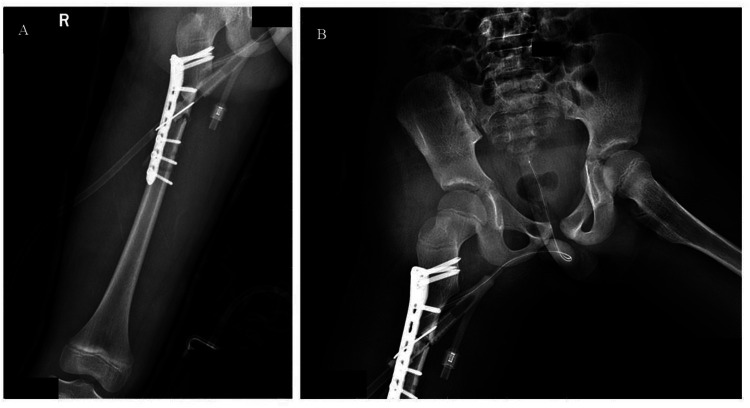
On postoperative day 3, radiograph demonstrates satisfactory alignment of the right femoral shaft fracture following internal fixation **(A)**, with the femoral head well-seated **(B)**.

**Figure 3:**
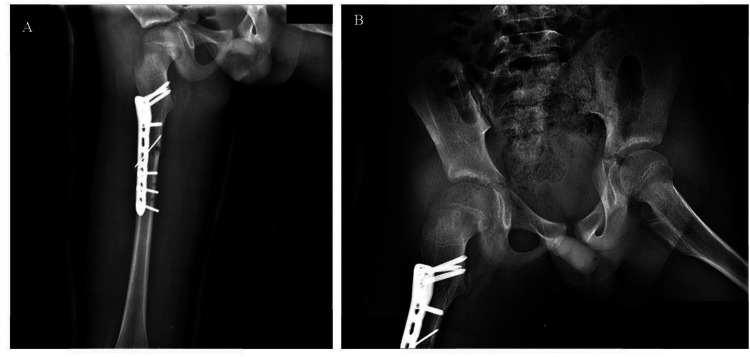
A x-ray demonstrated the healing of the right femoral shaft fracture at 1-month follow-up, with the right femoral head located in the acetabulum; B x-ray showed the right femoral head remains within the acetabulum on the 2-month follow-up.

**Figure 4:**
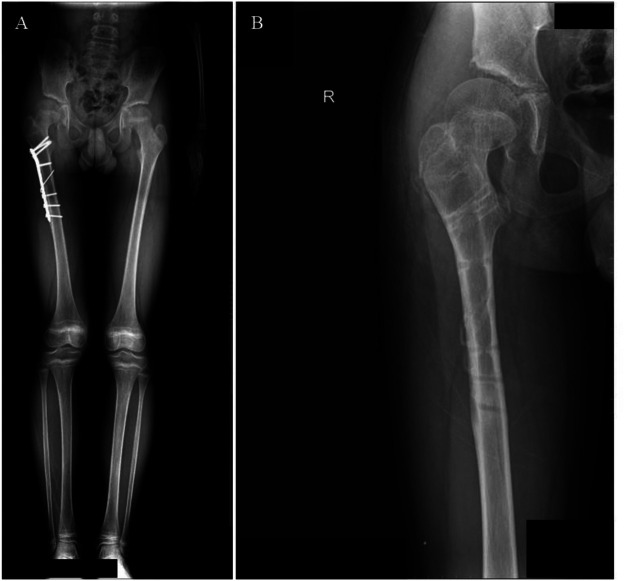
A full-length radiograph of both lower extremities, showed good healing of the right femoral shaft fracture at one-year follow-up, with the femoral head within the acetabulum and equal leg length; B demonstrated that after removal of the femoral shaft internal fixation, the right femoral head remains well-positioned with preserved morphology and no signs of AVN.

**Figure 5:**
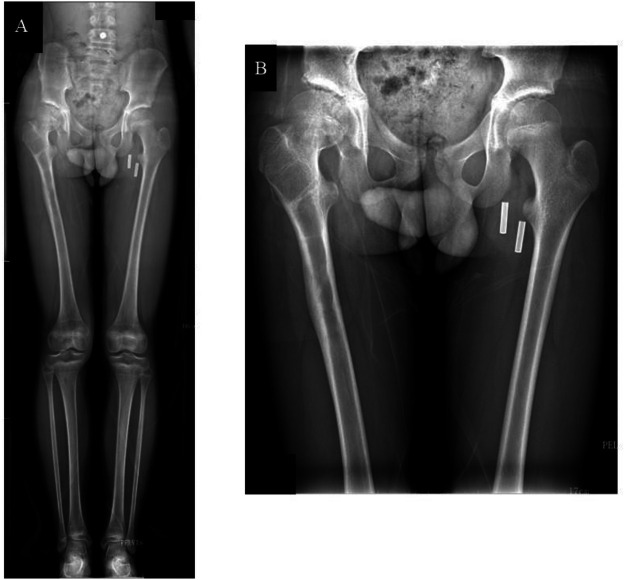
At the 1.5-year postoperative follow-up, full-length radiograph of both lower extremities **(A,B)** showed maintained equal leg length, well-remodeled union at the femoral fracture site, and preserved normal femoral head morphology with no signs of AVN.

**Figure 6:**
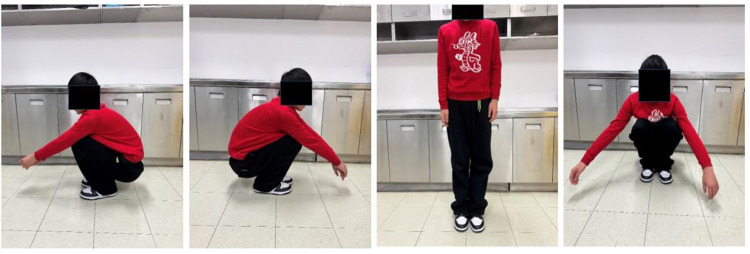
At 6-month follow-up after removal of the right femoral internal fixation, clinical photographs demonstrated satisfactory functional and cosmetic outcomes with equal leg lengths, and normal range of motion of the right hip.

At 18-month follow-up, the patient was pain-free with full hip range of motion (Figures 2–6). Radiographs confirmed femoral union, concentric hip reduction, and no AVN or leg-length discrepancy. Harris Hip Score was 98, indicating excellent function.

We acknowledge that 18 months may be insufficient for definitive AVN exclusion in children, where osteonecrosis can manifest up to 5 years post-injury. Long-term follow-up is planned at 3, 5, and 10 years with annual evaluation.

## Discussion

### The biomechanical challenge of pediatric “floating Hip” variant

Traumatic posterior hip dislocation with ipsilateral femoral shaft fracture is exceptionally rare in children and often labeled a “floating hip” variant (2, 15). Children's acetabula are elastic, making isolated dislocations uncommon, but high-energy trauma like motor vehicle accidents can cause this dual injury. The key challenge is the lost distal femoral lever arm: standard closed reductions (e.g., Allis or Stimson) rely on an intact femur for traction and torque. A fractured shaft leaves the proximal fragment unstable, risking physeal damage with forceful maneuvers (15, 16). Risk factors for poor outcomes include delayed diagnosis, older age, and high-energy mechanisms (11, 16).

To contextualize our case, we conducted a systematic review of ipsilateral hip dislocation with femoral shaft fracture (2010–2026), focusing on pure dislocation-shaft patterns without concomitant acetabular or femoral head fractures. Search strategy: PubMed, Embase, and Cochrane Library (January 2010–March 2026). Search terms: (“hip dislocation”[MeSH] OR “femoral head dislocation”) AND (“femoral shaft fracture”[MeSH] OR “femur fracture”) AND (“ipsilateral” OR “floating hip”) AND (“child”[MeSH] OR “pediatric” OR “adolescent” OR “adult”). Inclusion criteria: case reports/series describing pure hip dislocation with ipsilateral femoral shaft fracture; exclusion: combined femoral head fractures, significant acetabular fractures, or pathological fractures.

Only 3 pediatric cases (≤14 years) were identified in 16 years: two 11-year-olds (15, 17) and one 7-year-old (18)—all involving anterior (obturator) dislocations. This rarity necessitates comparison with adult patterns. Table 1 presents these pediatric cases plus our own; Table 2 provides adult/young adult reference. Outcomes emphasize the importance of prompt intervention, with AVN occurring in one delayed case (10 h post-injury) (18), while timely reductions showed no AVN (2, 8, 17). Common themes include the use of fixator-assisted closed reductions to restore leverage (8, 15, 16), with intramedullary nailing preferred for shaft fixation, aligning with adult patterns but adapted for pediatric growth plates.

**Table 1:** Literature review of “floating hip” variant: hip dislocation with ipsilateral femoral shaft fracture in pediatric patients (2010–2026)

Year	Authors	Age/Sex	Injury type	Treatment	Outcome	Approach	AVN
2024	Vatsyan et al.	11y/M	Posterior hip dislocation+ipsilateral femoral shaft fracture	External fixator pins for closed hip reduction+external fixator for shaft	Congruent reduction achieved; good early outcome	Fixator-assisted closed reduction	Not menthioned
2019	Cao et al.	7y/M	Anterior hip dislocation+bilateral femoral shaft fractures (right side associated with dislocation)	Closed/open reduction of hip at 10 hours+ORIF with plates/screws for both femurs	Fractures healed at 3–6 months; hardware removal at 6 months	Open for right shaft/left proximal; closed for left	Present (radiographic irregularities at 6 months indicating early AVN)
2016	Arjun et al	11y/M	Obturator (anterior) hip dislocation+ipsilateral femoral shaft fracture	Closed reduction of hip+intramedullary interlocking nail for femur	Fracture united at 6 months; full weight-bearing; near-normal hip ROM	Closed reduction of hip	Not mentioned

AVN, avascular necrosis; ROM, range of motion; ORIF, open reduction and internal fixation.

**Table 2:** Literature review of “floating hip” variant: hip dislocation with ipsilateral femoral shaft fracture in young adult patients (2010–2026)

Year	Authors	Age/Sex	Injury type	Treatment	Outcome	Approach	AVN
2024	Wuhib G, et al.	20 y/M	Anterior hip dislocation + ipsilateral subtrochanteric femoral fracture	Open reduction using Schanz pin traction + antegrade intramedullary nailing	Good functional recovery; full weight-bearing at 4 weeks	anterolateral approach	NO AVN
2020	Iftekharet al	24y/M	Posterior hip dislocation + ipsilateral femoral shaft fracture	Temporary external fixator for reduction + intramedullary nailing of shaft	Successful reduction and union	Closed reduction assisted by temporary external fixator	Not mentioned
2019	Rana et al.	18y/M	Posterior hip dislocation + ipsilateral femoral shaft fracture	Temporary external fixator for femur + closed hip reduction, followed by interlocking intramedullary nail	Successful reduction and fixation	Temporary fixator-assisted closed reduction	Not mentioned

AVN, avascular necrosis.

### Rethinking the surgical approach: why we chose an “untouched” anterior route

Our case's unique feature was using the anterior S–P approach for a posterior dislocation, diverging from the conventional posterior Kocher–Langenbeck method. In children, prioritizing biologic preservation is key, as anterior approaches minimize soft tissue injury and preserve vessels and nerves (19).

Posterior dislocations often tear the posterior capsule and short external rotators, endangering the medial circumflex femoral artery (MCFA), the primary pediatric femoral head supply. A posterior approach risks a “second hit” to residual vascularity through further dissection. The S–P approach, via the internervous plane (femoral and superior gluteal nerves), accesses the joint from an untouched anterior area, with modifications like avoiding rectus femoris detachment. This allowed hematoma evacuation, ligamentum teres excision, labral repair, and gentle reduction without posterior disruption. Literature shows anterior approaches effective for obturator dislocations (2, 17), with shorter operative times and less blood loss, though AVN risks rise with delays, as seen in one pediatric case (18).

### Surgical sequence: “femur first” to rebuild the lever arm

Sequencing in floating hip variant is debated (8). We fixed the femur first with a locking plate, then reduced the hip, aligning with staged fixation in mixed-age series (2, 8, 16, 17). This provided: (1) restored mechanical leverage for controlled reduction; and (2) stable positioning, reducing iatrogenic risks during capsulotomy. Literature supports femur-first in irreducible cases, often using external fixators or Schanz pins for leverage (2, 8, 15, 16), with intramedullary nailing common for shafts (2, 16, 17).

### Multifactorial AVN risk: beyond the 6-hour dogma

Conventional teaching emphasizes time to reduction as the paramount AVN determinant, with delays >12 h associated with >5-fold risk increase (6, 7). Our case—reduced at 25 h post-injury—prompts reconsideration of this paradigm. Multiple interacting factors modulate AVN risk: (1) trauma energy (high-velocity mechanisms cause more extensive vascular disruption); (2) concomitant fractures (femoral head or acetabular involvement directly compromises retinacular supply, absent in our pure dislocation-shaft pattern); (3) reduction technique (forceful closed manipulation risks physeal injury and vascular torsion vs. gentle open levering); (4) intraoperative vascular confirmation (drilling test, as performed, guides prognostic assessment); and (5) postoperative protection protocol.

The AVN-free outcome at 18 months despite delayed treatment suggests that meticulous vascular-preserving technique may partially compensate for temporal delays—specifically, anterior approach avoiding MCFA dissection, confirmed intraoperative perfusion, and biologically respectful reduction. This does not negate the imperative for urgent treatment when achievable, but offers prognostic nuance when delays are unavoidable. Long-term surveillance remains essential, as pediatric AVN may manifest up to 5 years post-injury.

Equally critical to long-term success is anatomic restoration of intra-articular structures, uniquely enabled by our anterior approach's direct visualization.

### Structural restoration via direct visualization

Direct visualization through the anterior approach revealed occult injuries invisible to closed methods: ruptured ligamentum teres, posteroinferior labral tear with buttonhole configuration, and capsular stripping. These mechanical obstacles—unaddressable by Schanz-pin joystick techniques (2)—required ligamentum teres excision, labral repair, and capsulorrhaphy, restoring joint congruency and suction seal. Unrepaired labral tears predispose to microinstability and secondary osteoarthritis (5); our anatomic repair, impossible through posterior or closed approaches, may mitigate long-term degenerative sequelae independent of AVN status.

### Surveillance protocol and outcome assessment

Despite favorable 18-month results, pediatric AVN may manifest up to 5 years post-injury. Current surveillance: radiographs at 3, 6, 12, 18 months; MRI at 24 months for occult AVN and labral healing assessment; annual evaluation thereafter to 5 years.

We acknowledge the absence of validated pediatric instruments (PODCI, PedsQL) and objective gait analysis as limitations to be addressed in long-term follow-up.

### Summary and clinical take-home

Pediatric floating hip variant management balances urgent reduction and vascular protection. Our anterior S-P approach with initial femoral fixation was effective, yielding excellent function without AVN. While not universal, this “fix femur first, approach hip anteriorly” strategy may be considered in select pediatric cases for optimal biologic and functional results, especially in high-energy trauma, as supported by diverse approaches in recent literature (2, 8, 15–18).

## Conclusion and clinical implications

For pediatric patients with this injury, anterior open reduction and rigid femoral fixation may be safe and effective in select cases, supporting anatomic reduction while potentially minimizing MCFA damage. Based on this single-case experience and limited pediatric literature (3 cases in 16 years), we propose that the strategy of femoral fixation preceding anterior open reduction maybe considered where: (a) closed reduction has failed; (b) posterior vascularity is of concern; and (c) intra-articular pathology requires direct visualization. This observation requires validation through multicenter collaboration given injury rarity. We emphasize that urgent reduction (<6 h) remains ideal, specialized pediatric trauma care optimizes outcomes, and long-term monitoring (≥5 years) is essential for AVN surveillance.

This case report was approved by the institutional ethics committee, and informed consent was obtained from the patient's guardian.

## Data Availability

The original contributions presented in the study are included in the article/Supplementary Material, further inquiries can be directed to the corresponding author.
